# Numerical Analysis of Microchannels Designed for Heat Sinks

**DOI:** 10.1007/s41871-021-00118-2

**Published:** 2021-11-11

**Authors:** Matthew McCormack, Fengzhou Fang, Jufan Zhang

**Affiliations:** 1 Centre of Micro/Nano Manufacturing Technology (MNMT-Dublin), School of Mechanical & Materials Engineering, University College Dublin, Dublin 4, Ireland; 2 State Key Laboratory of Precision Measuring Technology and Instruments, Laboratory of Micro/Nano Manufacturing Technology (MNMT), Tianjin University, Tianjin, 300072 China

**Keywords:** Microchannel, Heat sink, Conjugate heat transfer, Numerical simulation

## Abstract

Conjugate heat transfer is numerically investigated using a three-dimensional computational fluid dynamics approach in various microchannel geometries to identify a high-performance cooling method for piezoelectric ceramic stacks and spindle units in high-precision machines. Straight microchannels with rectangular cross sections are first considered, showing the performance limitations of decreasing the size of the microchannels, so other solutions are needed for high applied heat fluxes. Next, many microchannel designs, focusing on streamwise geometric variation, are compared to straight channels to assess their performances. Sinusoidally varying channels produce the highest heat transfer rates of those studied. Thus, their optimization is considered at a channel width and height of 35 and 100 μm, respectively. Heat transfer increases as the amplitude and spatial frequencies of the channels increase due to increased interfacial surface area and enhanced Dean flow. The highest performance efficiencies are observed at intermediate levels of amplitude and frequency, with efficiency decreasing as these geometric parameters are increased further at the onset of flow separation. The sinusoidal channel geometries are then optimized with respect to minimizing the system’s pressure drop for all applied heat fluxes between 5690 and 6510 kW/m^2^. Doing so created an optimal geometry curve and showed that all geometries in this region had amplitudes close to 40 μm. Therefore, imposing a fixed heat flux requirement for a case study of cooling piezoelectric ceramics, the optimized sinusoidal geometry decreases the system pressure drop by 79% relative to a straight channel while maintaining a larger minimum feature size.

## Introduction

The successes and rapid development in micro and nano manufacturing techniques have necessitated the need to engineer compact and high-performance miniaturized systems in several industrial sectors. Yet, the primary difficulty associated with the various applications of these technologies, such as computers, portable electronics, engines, high-precision machines, to name but a few, pertains to their heat management efficacy. In many applications, large-scale forced-air convection systems are unsuitable for the operating conditions or do not provide a high enough cooling flux. Therefore, many diverse micro-scale heat exchanging devices have been developed to meet these demands, including vapor chambers and heat pipes. Although they perform well, these devices have various disadvantages, including high cost, manufacturing complexity, and delicate and fragile configuration. A microchannel cooling system provides greater robustness and excellent heat transfer properties and thus is a more versatile option among these new solutions.

Microchannel cooling systems emerged in the early 1980s [1], following rapid developments in high-speed, high-power-density, and very-large-scale integrated circuits. These high-speed circuits dissipated thermal energy at a far greater rate than the capabilities of the small-scale cooling packages of the time, with systems limited to cooling rates of around 20 W/cm^2^. The compact, water-cooled microchannel heat sink developed by Tuckerman and Pease was tested at a power density of 790 W/cm^2^ [1].

Since this inaugural experiment, numerous studies have been conducted into microchannel cooling systems, both theoretical and experimental. With a large amount of associated design freedom, there are many different avenues for increased performance, such as the choice of coolant, solid material, and changes in microchannel geometry. However, this work is most interested in geometric change, as variations in material or coolant tend to be trivial (since the lowest thermal resistance should always be chosen for the highest heat transfer rate, and balancing the weighted benefits of these alterations is likely to be application-specific).

### Cross-Sectional Geometry

Traditionally, research was primarily focused on microchannels with rectangular cross sections, but advances in analytical, numerical, and manufacturing techniques have made it possible to study a wide variety of cross sections.

A pool of mainstream microchannel research is focused on the rectangular cross section due to its simplicity of analysis and manufacture. In an overview of microchannel research performed by Adham et al. [2], 57 of the 65 works reviewed used solely rectangular cross sections, though it must be noted that often these studies were not specifically interested in cross-sectional geometry. Most studies use a single fixed geometry that differs from peers’ research, despite cross section being shown to have a considerable impact on microchannel performance. These studies also employ a wide range of flow rates and applied heat fluxes. Thus, drawing parallels between different studies is challenging.

Li and Peterson [3] researched a wide variety of rectangular channel cross sections, ranging in width from 20 to 200 µm and height from 100 to 400 µm. This allowed for a range of 40–140 channels per cm width, incrementally increasing by 20 channels per cm. The lowest thermal resistance was observed at 120 channels per cm. This indicates that at some point, an increasing channel number density has adverse effects on the performance of the heat sink. An optimum ratio between the microchannel width and center-to-center channel spacing was found to be 0.7. The heat transfer rate was seen to be proportional to the aspect ratio of height/width, meaning a tall but narrow microchannel is advantageous. Further work in this area, including the optimization of these rectangular channels, can be seen in studies such as Husain and Kim [4], Wang et al. [5], and Wang et al. [6].

The heat transfer capabilities of differing cross sections have also been investigated and compared in detail. Triangular and trapezoidal cross sections, for example, have been studied by Li et al. [7], leading to the conclusion that the trapezoidal cross section was the superior of the two. Similarly, hyper elliptical and regular polygonal cross sections were investigated by Tamayol and Bahrami [8]. Furthermore, hexagonal, circular, and rhombic cross sections have been studied by Alfaryjat et al. [9]. Finally, a study by Tilak and Patil [10] investigated and compared a wide variety of differing cross sections. A significant difference was observed in channel wall temperatures and convective heat transfer coefficients due to the differences in cross-sectional geometry. In general, the cross section can be varied to increase the heat transfer rate, but this is accompanied by an increased pressure drop, leading to an increased required pumping power that would ideally be minimized.

### Streamwise Geometric Variation

Although the cross-sectional area is an important factor in the performance of heat sinks, other geometric factors can also alter performance significantly. How the channels vary throughout their length, for example, is also seen to be an essential factor.

Chai et al. [11] demonstrated periodic variance in the cross section led to reduced thermal resistance, especially at higher pumping powers when comparing microchannels with uniform cross sections to two designs with periodically expanding and contracting cross sections.

Similar periodic designs have also been tested by Ghaedamini et al. [12], Chai et al. [13], Zhai et al. [14], and Xia et al. [15]. In general, these studies also observed an increase in heat transfer from the periodically varying microchannels with only slight increases in pressure drop. Ahmed et al. [16] investigated both the frequency and symmetries of these periodic designs. It was seen that an increase in the frequency of these expansions did not always lead to an increase in performance attributed to complex microscopic eddy interactions. Instead, a higher heat transfer rate was reported when geometries exhibited greater symmetry. The asymmetric motion of eddies typically resulted in lower heat transfer values, and thus, the highest heat transfer values were seen in channels with axially symmetric variances in geometry.

Similar microchannel designs have also been investigated by Sui et al. [17], who specifically investigated wavy microchannels. These channels keep a fixed cross section, instead of a periodically increasing and decreasing one, but their path in plan-view varied sinusoidally. These designs were tested with varying amplitudes and frequencies, with others having varying frequencies throughout their length. Heat transfer rates were observed to increase with increasing frequency. This effect was attributed to the formation of Dean vortices, leading to chaotic advection, enhancing the convective heat transfer in the microchannel. It was also noted that the amplitude and frequency of this process could be varied along the length of the channels to cool possible regions of higher heat flux appropriately, mitigating hot spots. However, like other designs, a noted pressure drop penalty was associated with the design and increased with frequency.

Wavy microchannels have been further considered in research by Mohammed et al. [18], Gong et al. [19, 20], Rostami et al. [21], and Foo et al. [22]. In general, these structures have been researched. Yet, little attempt has been made to compare their performance to other commonly seen design options or to optimally choose geometric parameters over a wide range of operating conditions.

### Additional Geometric Features

Since the introduction of vortex generators in macroscopic systems to increase heat transfer performance by Johnson and Joubert [23] in 1969, numerous studies have also implemented vortex generators into microchannel systems. Liu et al. [24] experimentally investigated longitudinal vortex generators at various angle attack inflows of different Reynolds numbers. It was clearly shown that the critical Reynolds number for turbulent flow was decreased significantly from approximately 2300 down to 600–730 when the vortex generators were used. The heat transfer performance increased in laminar flows by 9%–21% and 39%–90% in turbulent flows. These had significantly associated pressure drop drawbacks. Turbulent structures were also noted far lower than typical Reynolds numbers for internal channel flow by Chen et al. [25].

The arrangement and angle of attack of these longitudinal vortex generators have also been investigated, for example, in the work of Datta et al. [26] or Zhang et al. [27]. Generally, the optimum angle of attack to a greater extent depends on the Reynolds number, with a decreased angle of attack becoming more suitable at higher Reynolds numbers.

### Research Question

A wide variety of suggested design improvements have been made to microchannels since their inception. Still, nearly all research resides in a low volume of design options compared to a straight rectangular microchannel. Although this provides an easy comparison to this typical design baseline, nearly all studies use a wide range of boundary conditions, geometric feature sizes, and differ in coolant and solid material choice, leading to a nearly impossible comparison between the designs proposed in individual research papers. If any of these designs were to be considered for use in any given application, it would be difficult to compare these designs; thus, extensive research would be required to address the issue, perhaps excluding simple variances in cross-sectional geometry.

With precisely that in mind, the purpose of this research is to investigate a wide variety of possible microchannel designs, identifying those designs that provide the highest heat transfer rate, and then examine these designs in greater depth, optimizing their geometry to suit specific applications. These designs are first considered in generality but are also more deeply considered for piezoelectric ceramic stack cooling applications. This application differs from much existing research as these systems can usually achieve higher Reynolds number flows than is typical, affording higher pressure drops since they are not as strictly constrained regarding pump sizes.

## Methodology

### Governing Equations, Problem Statement, and Simulation Setup

The analysis of the microchannel designs is investigated numerically using three-dimensional conjugate heat transfer computational fluid dynamics using *OpenFOAM*. This allows for the numerical computation of the flow field variables and the computation of the heat transfer rates and temperature field within the system's solid and fluid domains. This computational approach allows for the comparison of a wide variety of designs. In addition, the large data sets generated by this technique also allow for a thorough comparison of these different designs.

#### Governing Equations and Their Discretization

As this research primarily focuses on optimizing the channel geometry, the working fluid is set as water for simplicity. Considering the operating conditions, the flow can be assumed to be incompressible. Assuming water is a Newtonian fluid and ignoring contributions due to gravity leads to the following form of the continuity and momentum equations in differential form:

1 $$\nabla \cdot {{u}} = 0$$

2 $$\rho \left( {\partial_{t} {{u}} + \left( {{{u}} \cdot \nabla } \right){{u}}} \right) = - \nabla P + \mu \nabla^{2} {{u}}$$

where ${\varvec{u}}\left( {t,{\varvec{x}}} \right)$ is the velocity field, $P\left( {t,{\varvec{x}}} \right)$ is the pressure field, *μ* is the dynamic viscosity of the fluid, and *ρ* is the density of the fluid.

As the flow is considered to be incompressible, the energy equation becomes decoupled from the momentum equation in the fluid domain:

3 $$\rho C_{v} \left( {\partial_{t} T + {{u}} \cdot \nabla T} \right) = k\nabla^{2} T$$

where $T\left( {t,{\varvec{x}}} \right)$ is the temperature field, *k* is the thermal conductivity of the fluid, ${{C}}_{{{v}}}$ is the specific heat capacity of the fluid at constant volume, and *ρ* is the fluid density.

The energy equation in the solid domain is given by the heat equation:

4 $$\rho C\partial_{t} T = k\nabla^{2} T$$

where, $T\left( {t,{\varvec{x}}} \right)$ is the temperature field within the solid domain, *ρ* is the density of the solid, ${{C}}$ is the specific heat capacity of the solid, and *k* is the thermal conductivity of the solid.

To solve this system of equations as a boundary value problem, the system is discretized using the finite volume method. The finite volume method considers an arbitrary conserved property. Thus, when its conservation equation is transformed using Gauss’ Divergence Theorem, it can be applied to a substantial number of control volumes divided throughout the problem domain.

The time-averaged equations form of the equations can then be discretized using a variety of different numerical schemes. In this analysis, the cell-limited least squares schemes are employed to solve the gradient terms, whereas the bounded Gauss upwind scheme is used to solve the divergence terms, and a Gauss linear limited corrected scheme is used for the Laplacian terms. Where applicable, smooth solvers are employed to solve for velocity, enthalpy, internal energy, turbulent kinetic energy, dissipation rate, and specific dissipation rate. In addition, geometric agglomerated algebraic multigrid solvers were used in solving for density and pressure terms, and the preconditioned conjugate gradient solver was used to solve for the enthalpy in the solid.

#### Problem Statement and Boundary Conditions

**Fig. 1:**
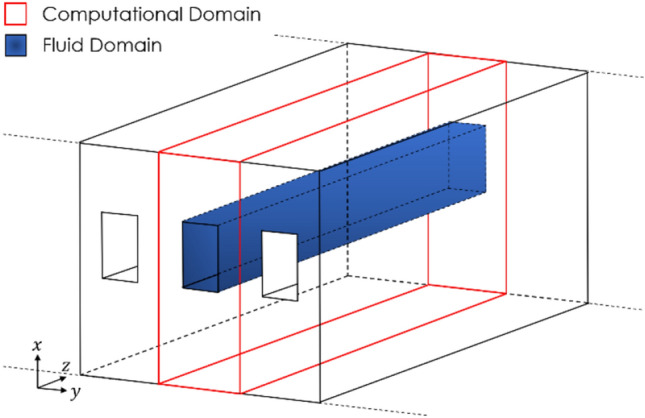
Schematic of a system of microchannels highlighting the computational domain

Regardless of the geometric changes investigated, the problem statement and boundary condition types stay the same. For example, a typical domain consists of many identical channels cut into a solid body. The domain can be reduced to a single channel to reduce the computational cost, as illustrated in Fig. 1, using a symmetry boundary condition on each dividing wall.

**Fig. 2:**
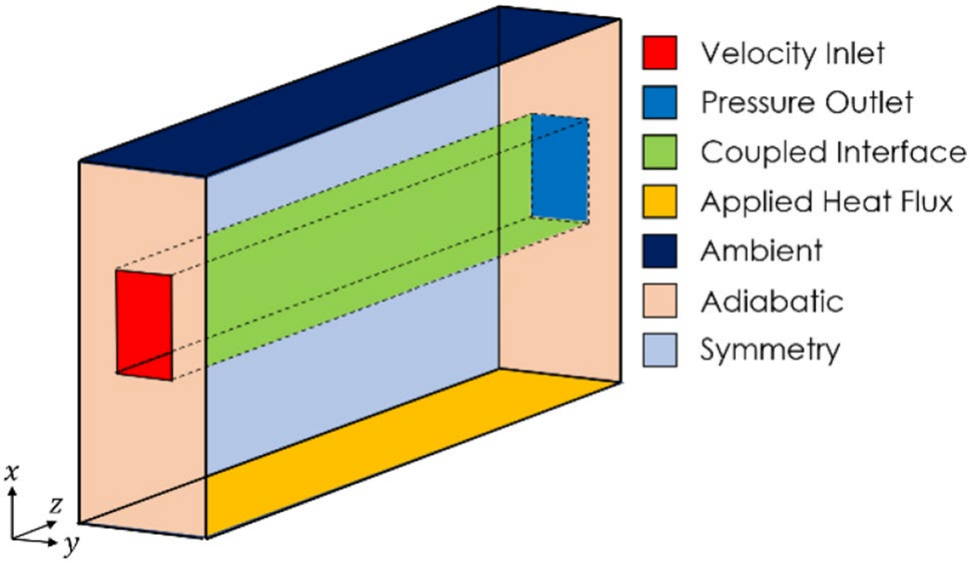
Boundary conditions of the computational domain

The channel is assumed to be heated from underneath, and thus a fixed flux heat source is applied to the bottom surface of the domain. The top surface of the domain is assumed to be accessible to ambient conditions. Thus a derived heat flux boundary condition is applied, specifying the ambient temperature of the room and a low convection coefficient. The inlet to the fluid domain is a uniform velocity inlet set to ambient temperature, and the outlet is a pressure outlet. Because the flow is assumed to be incompressible and thus the pressure is decoupled from the temperature, this pressure outlet serves only as a system reference pressure. The interfacial surfaces between the fluid and solid domains are designated as coupled interfaces, allowing energy to pass through them. The interfacial walls of the fluid domain are also set to have a no-slip condition to model viscous effects at the wall. Finally, the front and rear walls of the solid domain are set as adiabatic regions to ensure they do not contribute to the overall heat transfer, isolating the effects of the microchannel itself. These boundary conditions have been summarized in Fig. 2.

Since geometric changes are of most interest, the solid and working fluid properties are kept constant. These are shown in Table 1.

**Table 1:** Properties of the solid and fluid domains as used in the simulations and preceding calculations

Domain	Density, $\rho$(kg/m^3^)	Thermal conductivity, *k* (W/mK)	Specific heat capacity, ${\mathrm{C}}_{\mathrm{p}}$ (J/kg K)
Solid (copper)	8960	401	385
Fluid (water)	997	0.6	4180

#### Meshing

**Fig. 3:**
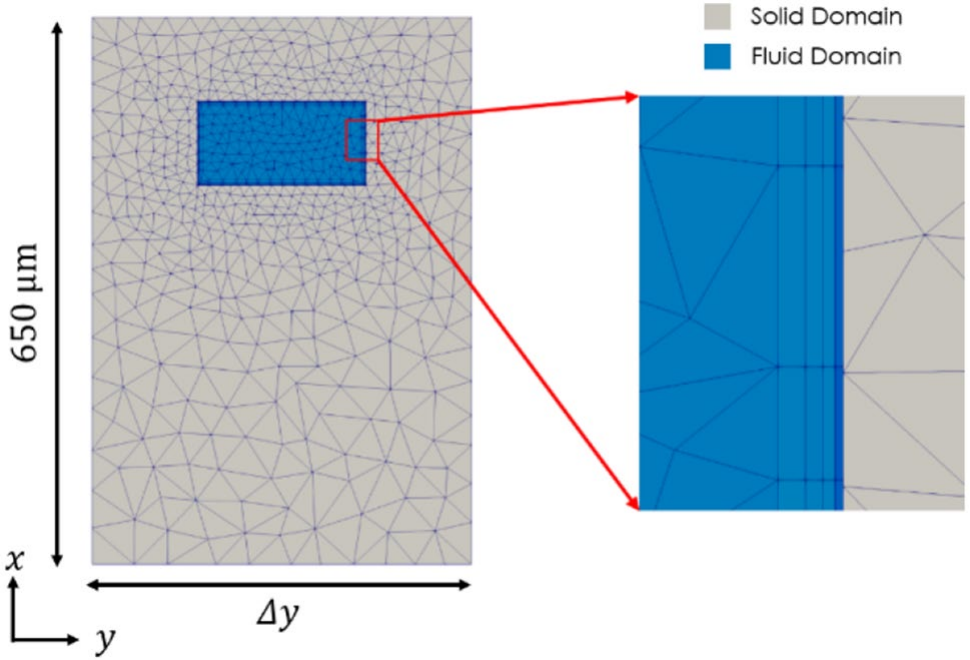
A typical computational mesh is shown at the front face of the geometry. The detailed view on the right shows the boundary layer cells and their growth from the wall

The domain mesh was formed using a tetrahedral and hexahedral hybrid mesh, where hexahedral cells were used to refine the boundary layer region at the walls of the fluid domain. The mesh is generated by choosing minimum specified edge lengths in both the fluid and solid domains. The boundary layer region is then specified, setting the initial layer’s relative thickness, number of layers, and growth rate. The growth rate in the solid region is then set such that the area of the cell face at either side of the coupled solid–fluid interface is approximately equal, allowing for better convergence with respect to energy transfer. The level of mesh refinement was iteratively increased for many different geometries until mesh independence was achieved. Deemed as such, the relative difference between mesh iterations of important system variables such as maximum temperature and maximum flow velocity and pressure was negligible. This level of mesh refinement was then applied to all subsequent simulations. The simulation's solution accuracy was also monitored using residuals of velocity, pressure, and convection coefficients, iterating the time-averaged solution until all of these residuals plateaued and remained at a minimum value. An average mesh for the domains discussed, all 14 mm in length, 650 μm in height, and widths $\Delta y$ ranging between [11.25, 450] μm, contained approximately 3 million computational cells. A typical cross section of a mesh, normal to the streamwise velocity component, is depicted in Fig. 3.

#### Data Collection

Time-averaged solution fields were analyzed using *ParaView*. This allowed for rendered images of solution fields and the exportation of numerical data sets to be evaluated in *MATLAB*. In monitoring the performance of each microchannel design, several standard values were recorded at constant locations in the geometry. These included temperature and velocity maxima and minima within the different subdomains and maximum and minimum pressures in the fluid domain to calculate the pressure drop across the system.

The rate of heat transfer to the coolant, $\dot{Q}$ is given by,

5 $$\dot{Q} = \dot{m}C_{p} \left( {T_{{f_{{{\text{out}}}} }} - T_{{f_{{{\text{in}}}} }} } \right)$$

where $\dot{m}$ is the mass flow rate and ${{C}}_{{p}}$ is the specific heat capacity at constant pressure. $T_{{f_{{{\text{in}}}} }}$ and $T_{{f_{{{\text{out}}}} }}$ are the fluid inlet and outlet temperatures, respectively.

The convective heat flux is given by,

6 $$\dot{q}_{{{\text{conv}}}} = \frac{{\dot{Q}}}{{A_{{{\text{conv}}}} }}$$

where ${{A}}_{{{\text{conv}}}}$ is the area over which convection takes place, i.e., the surface area of the channel walls.

The thermal resistance of the channel is,

7 $$R = \frac{{\Delta T_{\max } }}{{q_{{{\text{conv}}}} A_{b} }}$$

where ${\Delta T}_{{{\text{max}}}}$ is the maximum temperature difference between the solid bottom wall and the fluid and ${{A}}_{{b}}$ is the area of the base of the solid.

As is highlighted later in the Results and Discussion, comparing different performance measures that are normalized by area terms, such as the convective heat flux and thermal resistance, do not always correlate to the best performing microchannel, i.e., the channel which maintained the lowest temperature when subjected to the same applied heat flux source. This is due to the wide disparity in surface area values for the different geometries. In a similar sense, the heat transfer rate was also not a sufficient measure since this did not properly scale with the channel number density when analyzing the entire system. Thus, performance was measured mainly using a relative average base temperature decrease, defined for each geometry $j$ as follows:

8 $$(\overline{T}^{ + } )_{j} = \frac{{\overline{T}_{j,b} - \overline{T}_{{{\text{ref}},b}} }}{{\overline{T}_{{{\text{ref}},b}} - \overline{T}_{{{\text{ambient}}}} }} \times 100 {\%}$$

where $\overline{T }$ refers to the average temperature, the ${\text{ref}}$ subscript denotes the reference geometry for the study, and *b* denotes the property value at the base of the domain, i.e., the surface in contact with the heat source. In short, this value would express a percentage increase in performance as seen in a drop in average base temperature. When applied with the symmetry boundary conditions, this automatically scales to a system with many channels.

**Table 2:** Fixed dimensions and boundary conditions for the variation in channel width simulations

Channel length (mm)	Inlet velocity (m/s)	Inlet temperature (K)	Applied heat flux (W/m^2^)
14	10	293	250,000

The Reynolds number in this application is chosen to be:

9 $${\text{Re}} = \frac{{\rho UD_{H} }}{\mu }$$

where $D_{H}$ is the hydraulic diameter of the channel, and $U$ is the inlet velocity.

### Variation in Channel Width

As seen from Li and Peterson [3], a tall but narrow channel is advantageous in heat transfer, but to some degree, making the microchannel narrower results in a decreased performance. Precisely, microchannel widths were tested between 5 and 200 μm, keeping a fixed channel height of 100 μm and keeping the ratio between the channel width and the center-to-center channel spacing constant at a value of 4/9. Channel length was also fixed at 14 mm (Table 2). Therefore, testing this range of widths assesses the feasibility of modern manufacturing methods and their suitability to these devices within this operating window [28–31].

**Table 3:** Fixed dimensions and boundary conditions for the streamwise geometric variation simulations

Inlet cross section (mm × mm)	Channel length (mm)	Inlet velocity (m/s)	Inlet temperature (K)	Applied heat flux (W/m^2^)
0.2 × 0.1	14	10	293	250,000

A fixed flowrate was imposed on the system, leading to an inlet velocity of 10 m/s for all geometries. It can be seen from imposing continuity that the inlet velocity stays constant for the different geometries since neither the height of the channel nor the channel width to center-to-center channel spacing ratio changes with this change in width. Thus, as the width decreases, more channels would fit in the system, and the total channel cross-sectional area is conserved. The reference geometry for the average base temperature decrease, ${\overline{T} }^{+}$ was chosen to be a channel 200 μm in width. Considering the channel widths and flow rates, the Reynolds number for these simulations varies between 107 and 1498, and thus laminar flow can be assumed for these straight channels.

### Streamwise Geometric Variation and Other Features

A wide variety of different channel geometries appear in the literature, but few are compared to anything but a straight microchannel. Several standard channel classes are compared and discussed below, maintaining the channel width and height at 200 μm and 100 μm, respectively. The reference geometry in all cases is a straight rectangular geometry, 14 mm in length (Table 3).

**Fig. 4:**
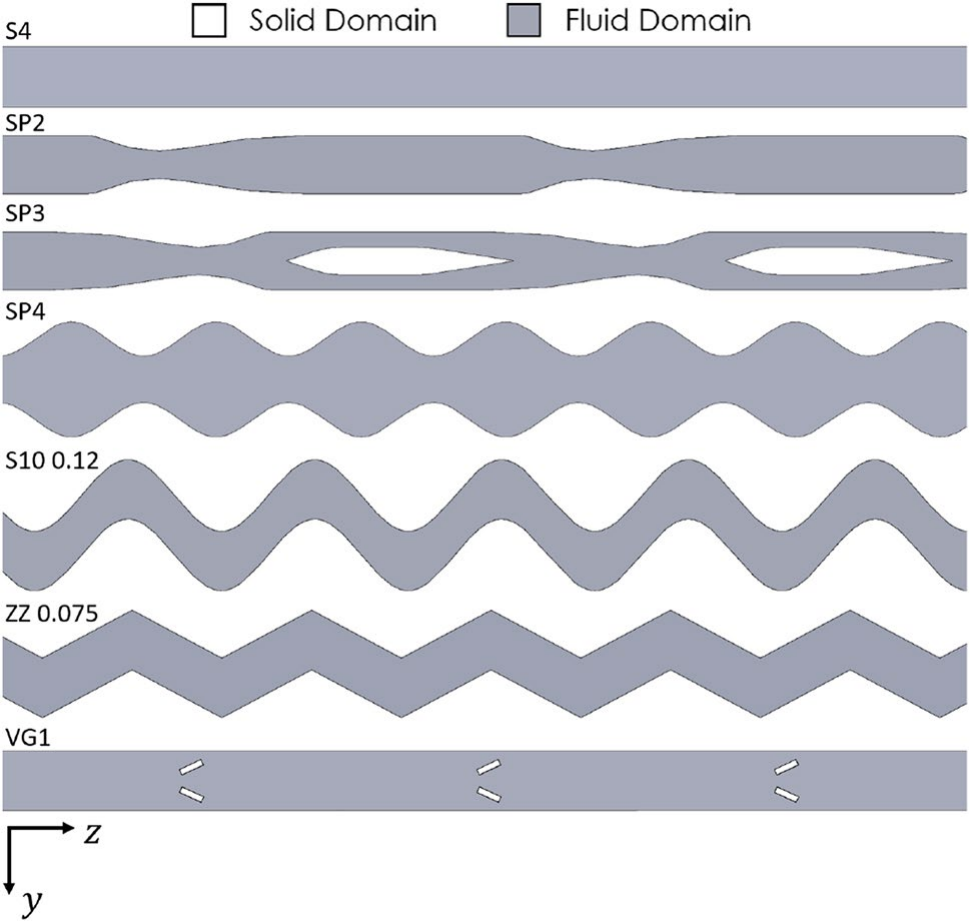
A selection of the channel geometries tested, showing the internal channel as represented by the fluid domain, labeled as per the descriptions of Sect. 2.3

1. Contraction and expansion channels
  These channels, which periodically expand and contract, have featured in several studies, including those of Chai et al. [11] and Ghaedamini et al. [12]. Additionally, geometric contractions and expansions outer walls and channel center were tested. This channel class has been denoted with an “SP” label in the results and is demonstrated in Fig. 4.
2. Sinusoidal channels
  These channels maintain a fixed channel width, with the entirety of the section varying periodically. Adjusting the amplitude and frequency of the channel results in an alteration of the flow fields and thus the heat transfer rate. These channels have been denoted by an “S*x*-*y*” label where *x* refers to the frequency and *y* refers to the sine wave’s amplitude. For example, “S10-0.075” represents a channel with a spatial frequency of 10 mm^−1^ and an amplitude of 0.075 mm. Similar channels are also tested but with sharper corners than the sinusoidal channel. These “zig-zag” channels are represented by a “ZZ *y*” label, where *y* is the channel's amplitude in mm. A sample of these geometries is shown in Fig. 4.
3. Vortex generators
  Although significant effort has gone into investigating vortex generators in microchannels, their performance impact relative to other types of geometric variation is of interest. Hence, a single vortex generator design was tested. The geometric parameters of the vortex generators were chosen to be close to those of Ebrahimi et al. [32]. These vortex generator geometries have been denoted by a “VG” label. An example of this is shown in Fig. 4.

The Reynolds number for these channels is approximately 1500. However, laminar flow cannot be assumed since turbulent structures have been noted experimentally at Reynolds numbers less than this in similar geometries [24, 25]. Thus the *k*-*ω* SST model has been used for these simulations.

### Sinusoidal Optimization

Following the results of Sect. 2.3, the sinusoidal channels were seen to have the most significant potential for high heat transfer rates, exhibiting the highest heat fluxes and lowest base temperatures of those geometries tested. Thus, the performance impact due to changing the sinusoid’s geometric parameters is of interest, and ultimately, the choice for these parameters is optimal for any given application.

To do so, minimizing the number of free variables and thus choosing to keep the width and height of the channels constant is preferential. These were selected as 35 μm and 100 μm, respectively. The analysis of Sect. 2.2 showed that the pressure drop increases significantly once the width of the channel drops below a value of approximately 25 μm. Thus the optimization is performed at 35 μm to gain further performance while avoiding the region of high-pressure growth. To keep the pressure drop reasonable and a high channel height to width ratio recommended by Li and Peterson [3], the height of the channel is fixed at 100 μm. Constraining these factors leads to a constant Reynolds number of 580 for all tested geometries, based on the hydraulic diameter of the channel, meaning laminar flow can be assumed.  These dimensions and boundary conditions are summarised in Table 4.

**Table 4:** Fixed dimensions and boundary conditions for the sinusoidal optimization simulations

Inlet cross section (mm × mm)	Channel length (mm)	Inlet velocity (m/s)	Inlet temperature (K)	Applied heat flux (W/m^2^)
0.035 × 0.1	14	10	293	250,000

The channel can be parameterized, with the wall of the channel being described by the function $F\left(z\right)=A \mathrm{sin}(fz)$, where *z* is the coordinate that runs along the length of the channel. The wavelength of this curve can be related to the spatial frequency *f* through $\lambda =2 \uppi /f$.

**Fig. 5:**
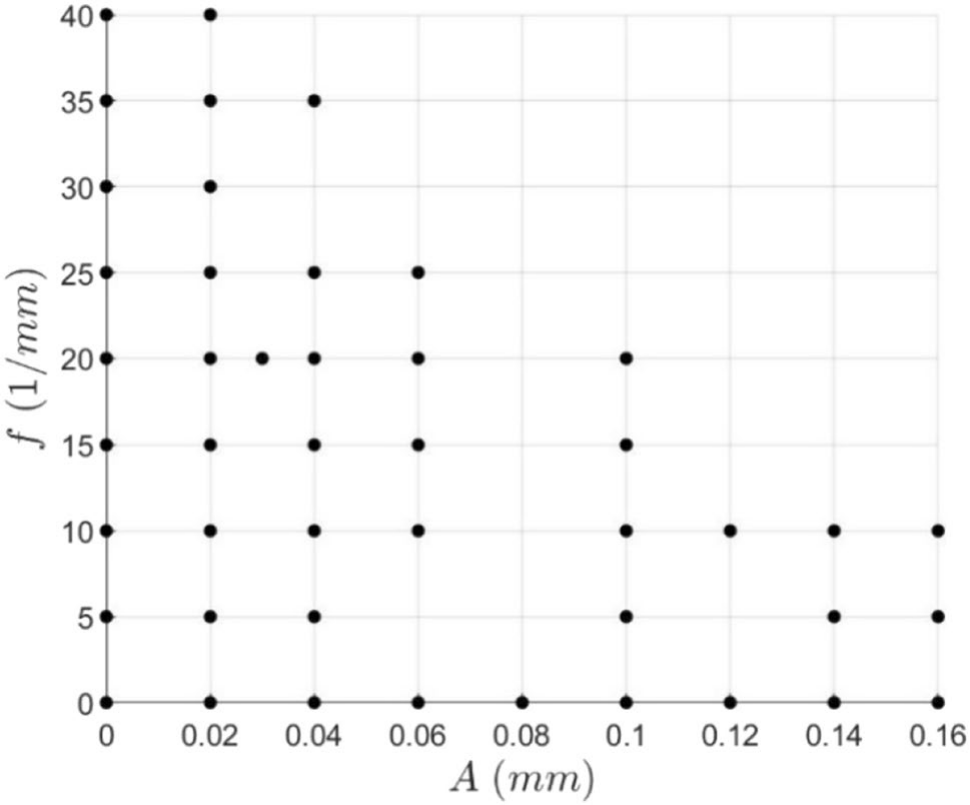
$\{A,f\}$ plane showing each of the geometries tested in the optimization. Each point $(A,f)$ represents a different geometry

**Fig. 6:**
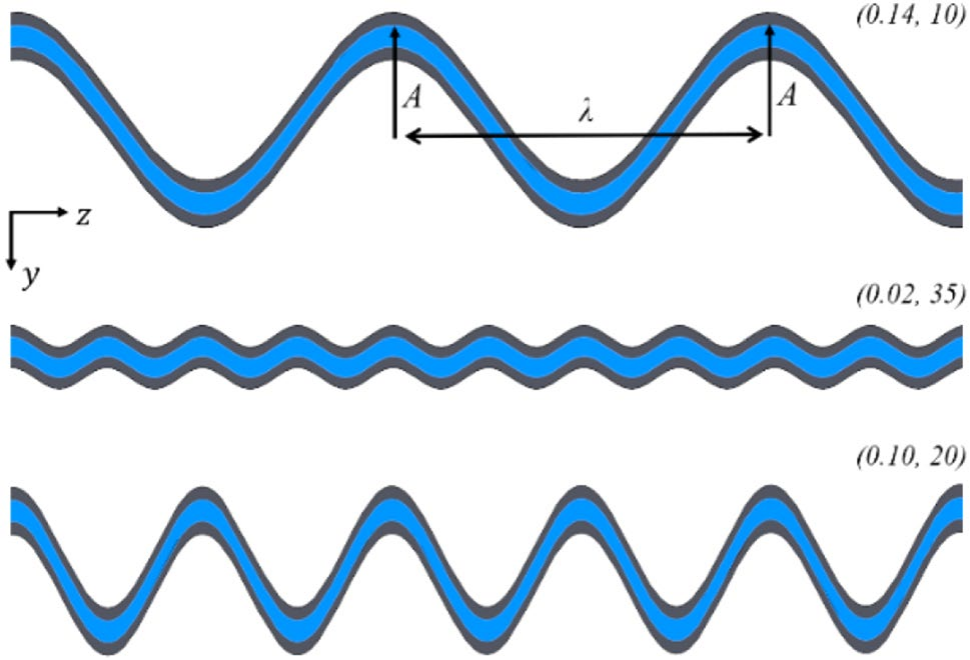
Internal geometry of a selection of tested channels, shown in cross section with approximately 10% of the channel length in view. Fluid domain (*blue*), solid domain (*gray*)

To achieve an optimization process, the values of certain performance variables, such as the average base temperatures and pressure drops, are derived as functions of the spatial frequency *f* and channel wall amplitude *A*. These properties can be thus described as a set of surface functions on an orthogonal basis $\langle A,f\rangle$ and thus by choosing a suitable discretization of the $\{A,f\}$ space, the problem is reduced to a series of surface interpolation problems. Here $\langle A,f\rangle$ is the natural basis choice for this surface interpolation problem. By examining the limits as *A* and *f* independently tend to zero, the channel wall function tends to a straight line of slope zero, i.e., the straight microchannel, which is the reference geometry. Thus, any geometry along the $A$ and $f$ axes is purely a straight microchannel, giving boundary conditions to the problem. Choices of particular geometry, referencing a single point $(A,f)$, can be chosen iteratively as the output variables of each simulation are monitored. So, by setting an upper allowable pressure drop limit on the system, the domain of interest is reduced to that shown by the extremities of the tested geometries in Fig. 5. This cut-off can be done since the pressure drop is expected to increase monotonically with increased $A$ and $f$, at least at relatively large values of $A$ and $f$. The specific value of this pressure drop cut-off was chosen to be close to that of a straight channel that produced similar heat transfer rates, albeit at a reduced channel width. This was done because a straight channel is likely desirable unless the sinusoidal channel can significantly improve performance. Geometries $(A,f)$ can then be chosen within this domain to achieve a desired level of smoothness in the surface interpolations. These surfaces can then be used to find an optimal geometry for a given application in multiple ways, two of which are considered in the results. The resultant geometries chosen through this process are shown in Fig. 5. A selection of these geometries in physical space are shown in Fig. 6 for reference.

In addition to taking a black-box approach by purely monitoring output variables, the flow fields are also considered to gain insight into the physics of the problem, attempting to correlate the features of the surface plots with the observed flow mechanisms.

## Results and Discussion

### Variation in Channel Width

**Fig. 7:**
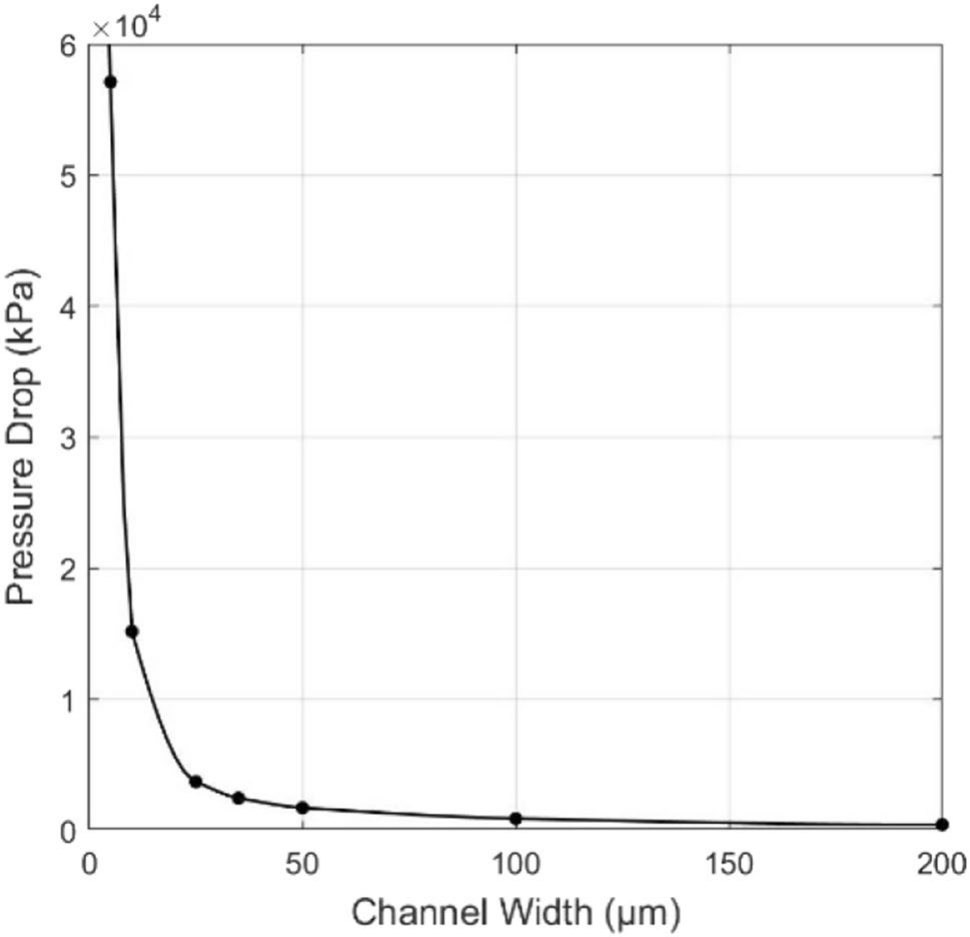
Pressure drop across the system at varying channel widths

**Fig. 8:**
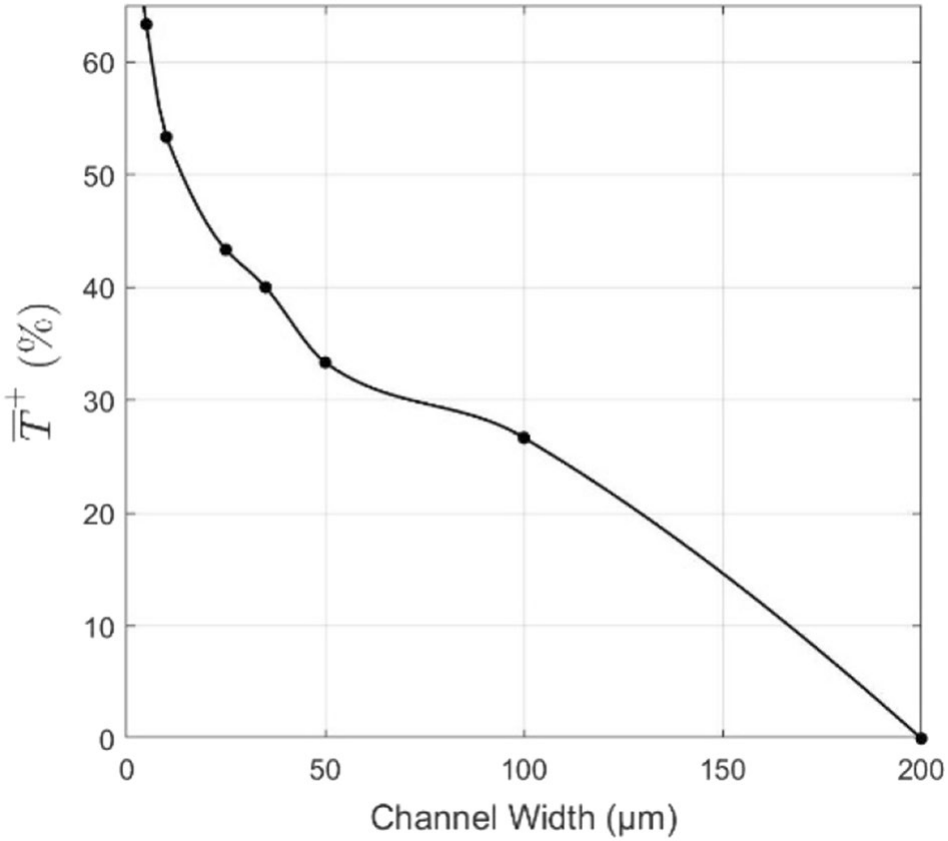
Average base temperature decrease at varying channel widths

The results shown in Figs. 7 and 8 quite clearly show that although the average base temperature continues to decrease as the width decreases, it is accompanied by a very rapid increase in the pressure drop as the width drops below around 25 μm. This is a key finding, as for applications with extremely high cooling requirements (Table 5), purely decreasing the channel size is insufficient as a means to keep increasing the heat transfer rate. This indicates that choosing the smallest channel width is not necessarily beneficial if smaller feature sizes can be achieved. Thus, it creates an opportunity for performance gains using other geometric features such as vortex generators.

**Table 5:** Numerical results of the channel width variation simulations

Channel width (μm)	Reynolds number	Avg. base temperature (K)	${\dot{{q}}}_{\mathrm{base}}$(kW/m^2^)	Pressure drop (kPa)
200	1498	296	212	530.414
100	1124	295.2	229	994.523
50	750	295	234	1,815.865
35	583	294.8	238	2,553.361
25	449	294.7	241	3,807.584
10	204	294.4	247	15,287.55
5	107	294.1	144	57,216.68

### Streamwise Geometric Variation and Other Geometric Features

Comparing the results of the simulations, it is clear that there are discrepancies between the different performance measures shown in Table 6. With this in mind, it is assumed that the system's goal is to minimize the temperature of the heat source, i.e., the temperature of the medium assumed to be in contact with the base of the domain. Thus, the average base temperature is seen as the primary indicator of performance. The results show how measures normalized by surface areas can be misleading. As changes in geometry occur, multiple geometries exhibited higher heat fluxes or lower thermal resistances than the sinusoidal and zig-zag channels, which were the channels that obtained the lowest average base temperatures. This is important to note, as many studies do not use temperature-based measures and rely solely on heat transfer rates or thermal resistances when analyzing the performance of microchannels.

**Table 6:** Numerical results of the streamwise geometric variation simulations

Geometry	Avg. base temp. (K)	$\dot{{Q}}$(W)	${\dot{q}}_\text{conv}$(W/m^2^)	${R}$(K/W)	Pressure drop (kPa)
S0.2 × 0.1	296	1.333	158,690	3.745	222.37
SP1	295.9	1.359	165,328	8.092	668.97
SP2	295.6	1.417	179,822	6.729	969.71
SP3	295.3	1.367	188,292	6.705	1747.48
SP4	296.3	1.717	132,689	3.699	454.24
S10-0.075	295.2	1.467	167,466	2.891	1697.17
S10-0.12	294.9	1.525	164,534	2.605	3670.76
ZZ 0.075	295.2	1.55	176,941	2.436	2791.15
VG 1	295.4	1.381	164,515	7.283	1829.60

**Fig. 9:**
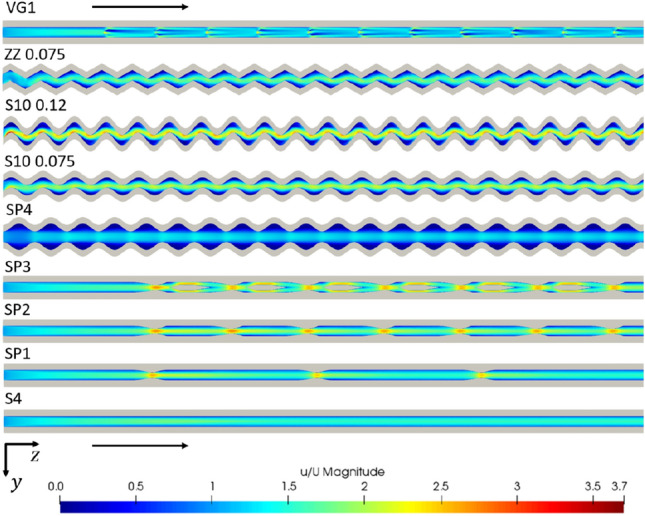
Scalar velocity field normalized by the inlet velocity shown for each tested geometry, viewed in cross section from above. Flow moves from left to right as indicated by the* arrows*. Channel length: 14 mm. Re = 1500

**Fig. 10:**
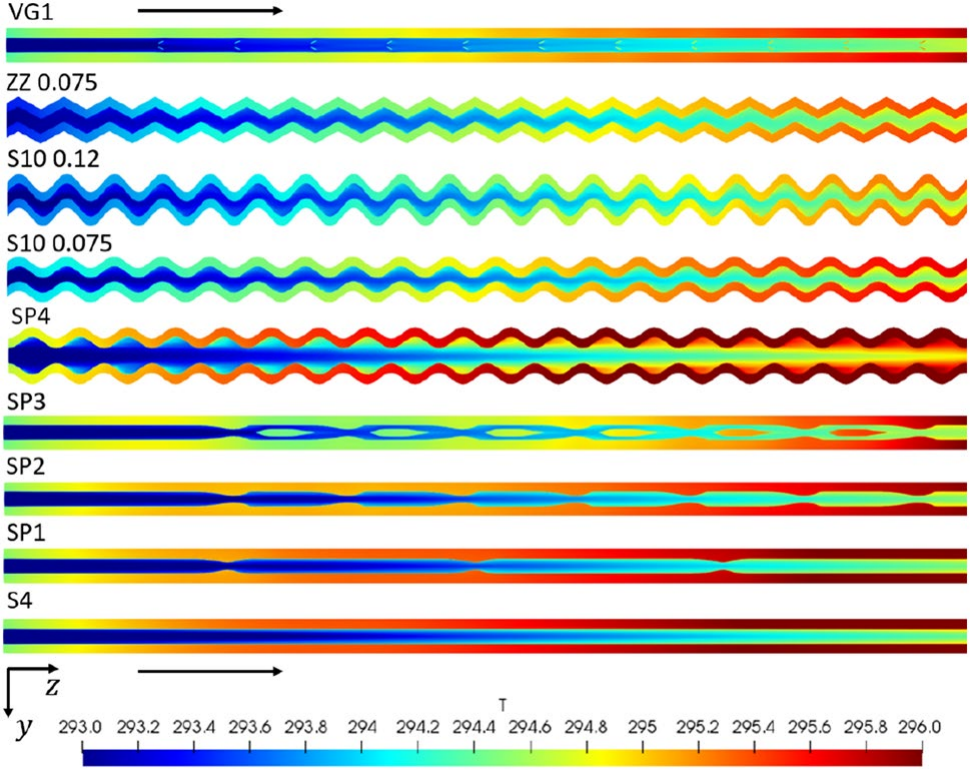
The temperature field is shown for each of the tested geometries, viewed in cross section from above. Flow moves from left to right as indicated by the* arrows*. Channel length: 14 mm, Re = 1500

Figures 9 and 10 show the velocity and temperature fields within a cross section of each channel. The temperature of the fluid and surrounding solid, in particular, can be easily compared between different geometries. Notably, the channel wall temperature varies significantly between channels, with the sinusoidal channels having the coldest channel wall temperatures.

**Fig. 11:**
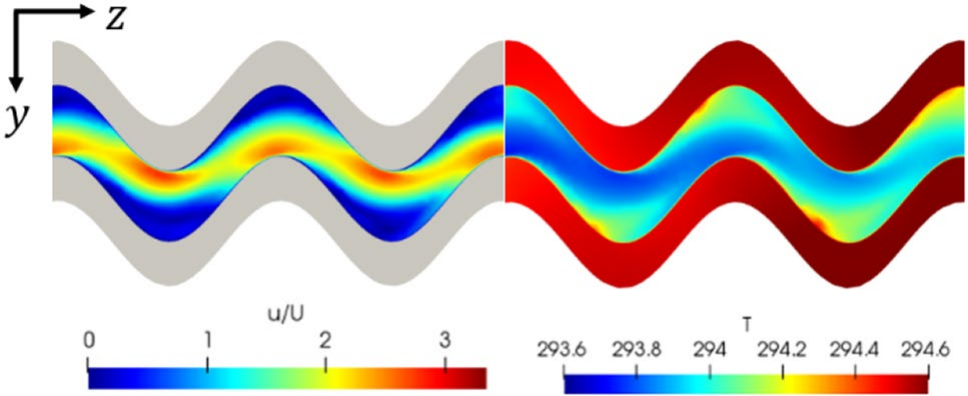
Velocity (normalized by the inlet velocity)/ temperature of the fluid shown centered at *x*/*L* = 0.07 for geometry S10-0.12, viewed in cross section from above (flow from left to right). Re = 1500

Sinusoidal channels were seen to show the highest heat transfer performance exhibiting low average base temperatures and low thermal resistances relative to the other geometries tested (Table 6). In particular, the relative average base temperature decrease, ${\overline{T} }^{+}$ increased by around 36% for the best performance of these geometries relative to the straight microchannel reference (Eq. 8). They did, however, indicate a high-pressure drop relative to the straight channel. Increasing the sinusoid amplitude was seen to lower the average base temperature and increase the pressure drop further. Superior heat transfer can be attributed to an increased convective surface area and a higher thermal energy diffusion rate observed in the concave regions near the channel wall. This higher thermal diffusion rate is evident in examining Fig. 11, where an increased temperature is observed in the large separation regions of low-velocity fluid. This low-velocity, high-temperature fluid is then forced to mix with the high-velocity stream by the sinusoidal geometry. Zig-zag channels were seen to produce similar performance levels as the sinusoidal channels, outlined by the average base temperatures in Table 6, but at an increased pressure drop due to the sharpened extrema.

Expansion and contraction geometries lowered the average base temperature relative to the straight geometry. However, the pressure drop increased moderately compared to that of the sinusoidal geometries, and thus this channel class may be best in applications where lower applied heat fluxes exist. A disadvantage of these geometries is that the inlet cross section must typically be sizable compared to other channel classes for fixed minimum feature size, leading to a relative reduction in performance. This is clear when applied to design SP4. Here the average base temperature was higher than that of the rectangular design, but the pressure drop was also decreased relative to other channels.

**Fig. 12:**
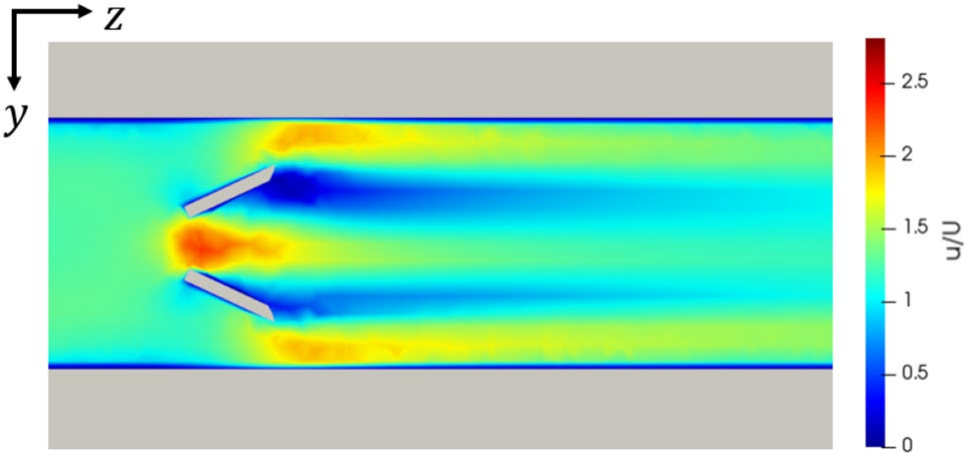
Wake of vortex generators as used in design VG1. Channel width: 0.2 mm, Re = 1500

The use of vortex generators decreased the average base temperature, but a significant pressure drop was incurred relative to the increased heat transfer. Large separation regions manifested behind the vortex generators, as indicated in Fig. 12, and these regions distorted the temperature field within the channel as intended. The heat transfer was increased relative to the straight channel by an amount similar to the experimental readings of Liu et al. [24], with the average base temperature decreasing by around 20%. The pressure drop appeared greater than predicted by the work of Ebrahimi et al. [32]. Therefore, this is indicative of the performance sensitivity of these devices relative to their operating conditions.

At the working conditions of interest, the sinusoidal channels are seen to perform the best of the designs tested. These channels also have the distinct advantage of minimizing the width of the channel for a given minimum feature size. The large pressure drops associated with these geometries can be reduced by increasing the channel's height or decreasing the sinusoid's amplitude. The amplitude and frequency of the sinusoid need not be constant throughout the length of the channel and thus can be varied to address hot spots. Varying the amplitude and frequency throughout the channel’s length could also be used to increase cooling performance near the exit of the channel where the temperature difference between the coolant and solid has decreased.

### Sinusoidal Optimization

**Fig. 13:**
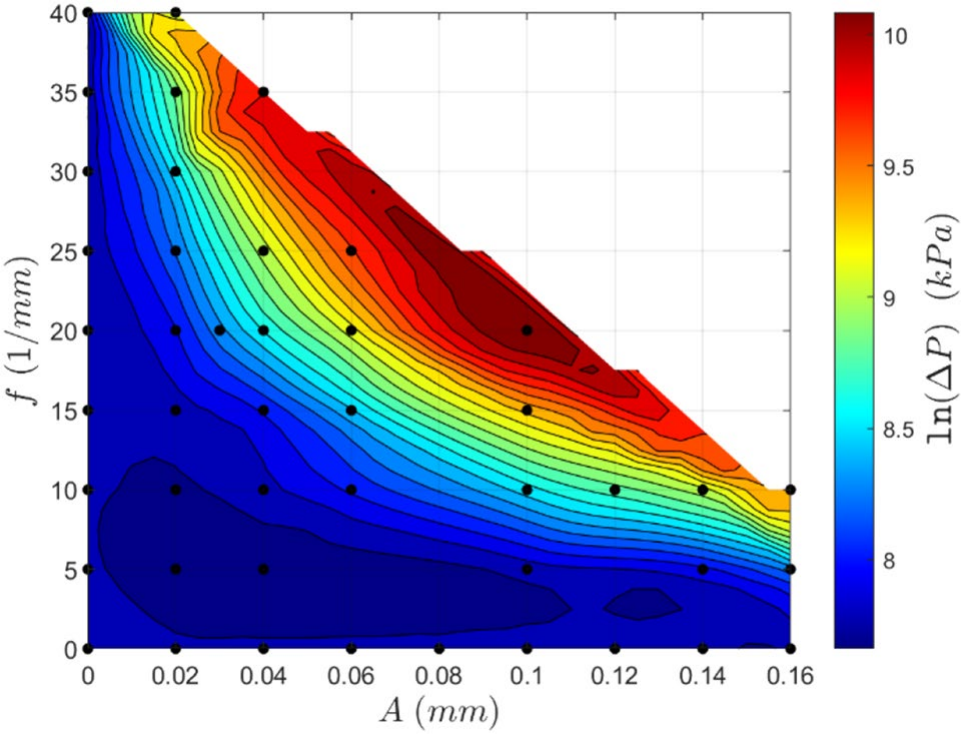
Contour plot of the natural logarithm of the pressure drop for a variety of geometries. Re = 580

Examining the contour plots of the surface interpolations, the general trends for the pressure drop and the average base temperature decrease are observed. In general, the pressure drop increases with increased frequency and amplitude, with peak pressure drops incurred when both amplitude and frequency increase (Fig. 13). That being said, the pressure drop increase is quite reasonable in proximity to the axes, and thus, not straying too far from a straight channel appears to be desirable.

**Fig. 14:**
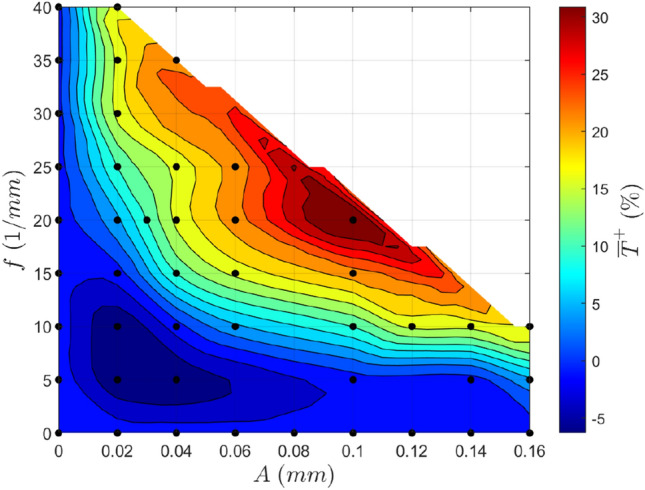
Contour plot of the avg. base temperature decrease ${\overline{T} }^{+}$ for a variety of geometries. Re = 580

A reasonably similar trend is observed for the average base temperature decrease (Fig. 14), with high-performing channels tending to correlate with a high-pressure drop. At fixed amplitude, the average base temperature decrease is seen to increase rapidly with increased frequency, excluding the region of poor performance at low frequencies and amplitudes, as shown in the dark blue area in Fig. 14. Notably, the average base temperature decrease begins to plateau with increased frequency at lower amplitudes (seen in amplitudes less than 0.06 mm), meaning that increasing the frequency becomes less effective to gain performance at higher frequencies incurring a relatively large increase in pressure drop. An increase in amplitude is seen to shift these fixed amplitude curves closer to the amplitude axis such that the steep increase in performance occurs at lower spatial frequencies. Similar trends are observed in the alternate case where curves of fixed frequency are examined.

In general, equivalent changes in frequency or amplitude result in a similar output, and thus, choosing an optimal geometry from only these outputs is complex. This means that other approaches must be used to seek a path for further development or determine an optimal geometry.

**Fig. 15:**
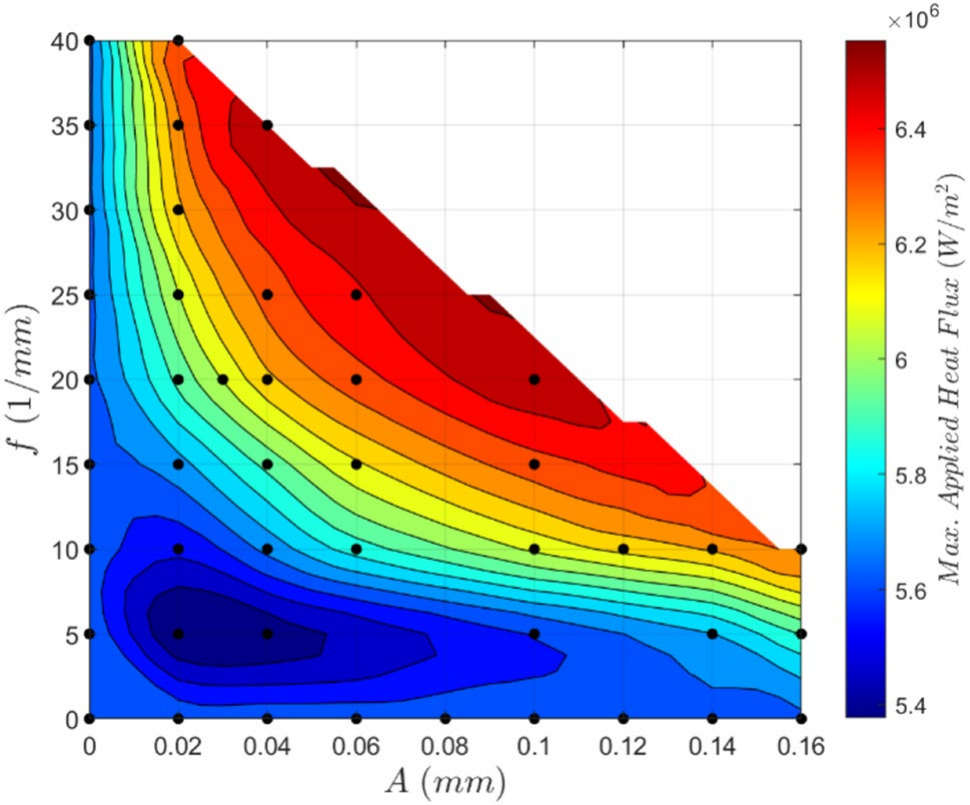
Contour plot of the maximum applied heat flux for a variety of geometries. Maximum allowable temperature: 80ºC. Re = 580

As well as examining temperature changes, we can also approach the problem from a heat flux perspective. Given an arbitrary application, one can likely calculate the heat flux requirements of the cooling system needed. Thus, it is helpful to know the maximum heat flux that can be applied to each geometry. To do this, one must first prescribe a maximum allowable temperature for the system. If an 80ºC cut-off is selected, then the maximum applied heat flux can be computed for each geometry. The surface interpolation of these values is shown as a contour plot in Fig. 15.

**Fig. 16:**
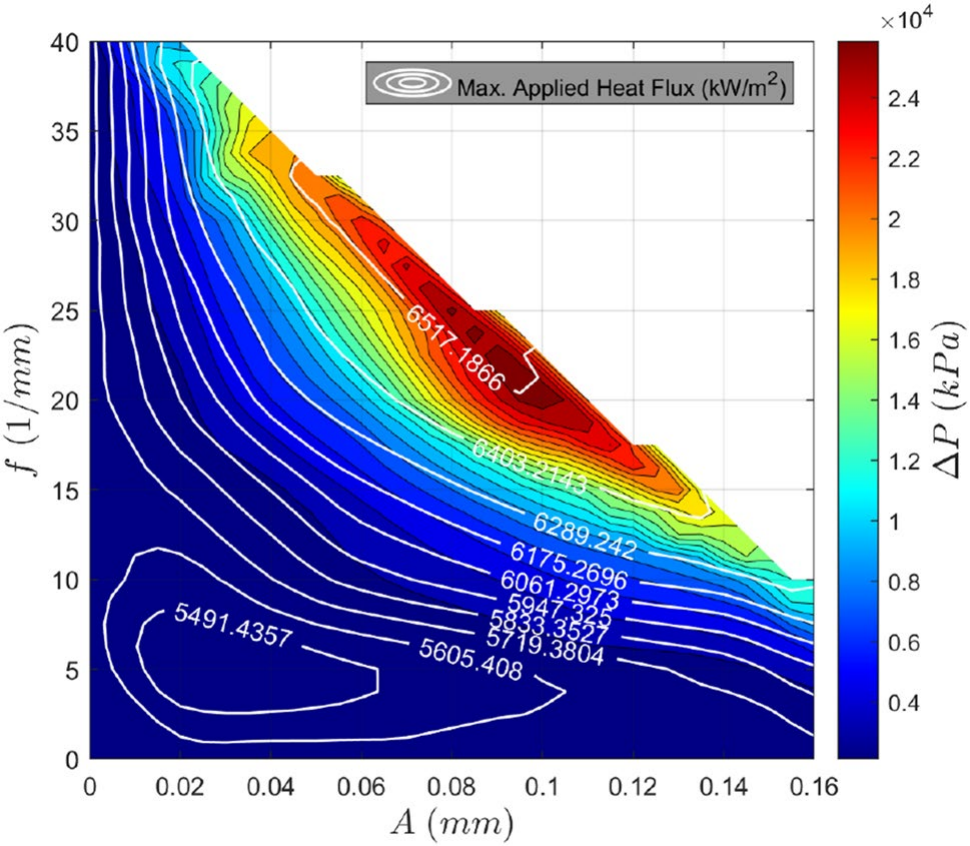
Generalized optimization plot. Maximum allowable temperature: 80 ºC. Re = 580

This maximum absorbable applied heat flux plot can be combined with the pressure drop contours to optimize for a given application. This is done by first calculating the heat flux emitted by the heat source, incorporating any desired safety factors. This heat flux would, in turn, correspond to a distinct contour on the maximum applied heat flux plot. This contour on the $\{A,f\}$ plane can then be mapped to the pressure drop contour plot. The highest performance design can then be determined by computing this heat flux contour mapping, searching for the lowest possible pressure drop. Doing so achieves the lowest possible pressure drop while ensuring the heat flux requirements are met. This process can also be accomplished visually by considering a generalized optimization plot shown in Fig. 16.

**Fig. 17:**
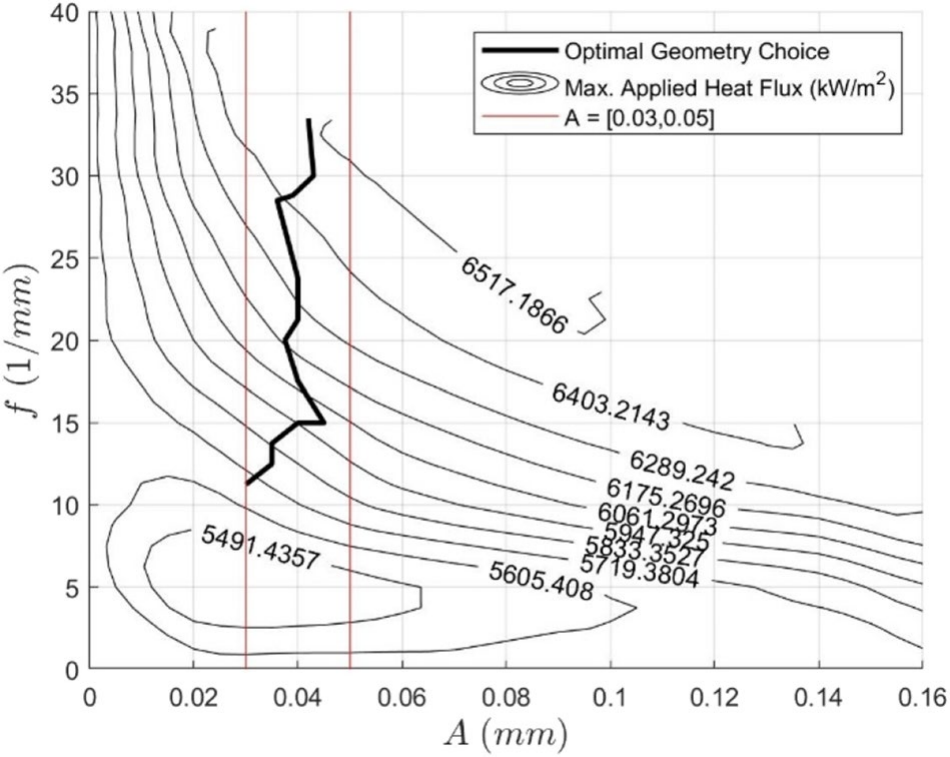
Optimal geometry choice for each maximum applied heat flux between 5690 and 6510 kW/m^2^ whereby the system pressure drop is minimized for each value. Maximum allowable temperature: 80ºC. Re = 580

If it is assumed that for each value of the maximum absorbable applied heat flux, there is a unique minimum for the pressure drop, then this process can be repeated for each heat flux in the domain to trace an optimal design curve in the $\{A,f\}$ plane. This is depicted in Fig. 17 and represents the best choice of (*A,f*) for each maximum absorbable applied heat flux between 5690 and 6510 kW/m^2^, as indicated by the contours. The optimal choice in this range tends to stay close to an amplitude of 0.04 mm as the frequency increases to increase the maximum heat flux. Given that there was no clear choice between varying amplitude and frequency based on the analysis of Figs. 13 and 14, this is an intriguing result.

When applied to a case study, improvements due to these sinusoidal channels can be considerable. For example, the discussed optimization strategy for sinusoidal channels, i.e., Fig. 17, was implemented to cool piezoelectric ceramics used in a nanomanufacturing method, where a known heat flux was emitted from the ceramics. Using this value as the maximum applied heat flux, the pressure drop was decreased by 79% due to using the optimal sinusoidal channel relative to the straight rectangular channel geometry, which produced the same heat flux. Importantly, this was also achieved with the sinusoidal channel having a larger minimum feature size, 35 μm, compared to the straight channels’ minimum feature size of 10 μm (this reduced minimum feature size of the straight channel was needed to be able to meet the heat flux requirement).

**Fig. 18:**
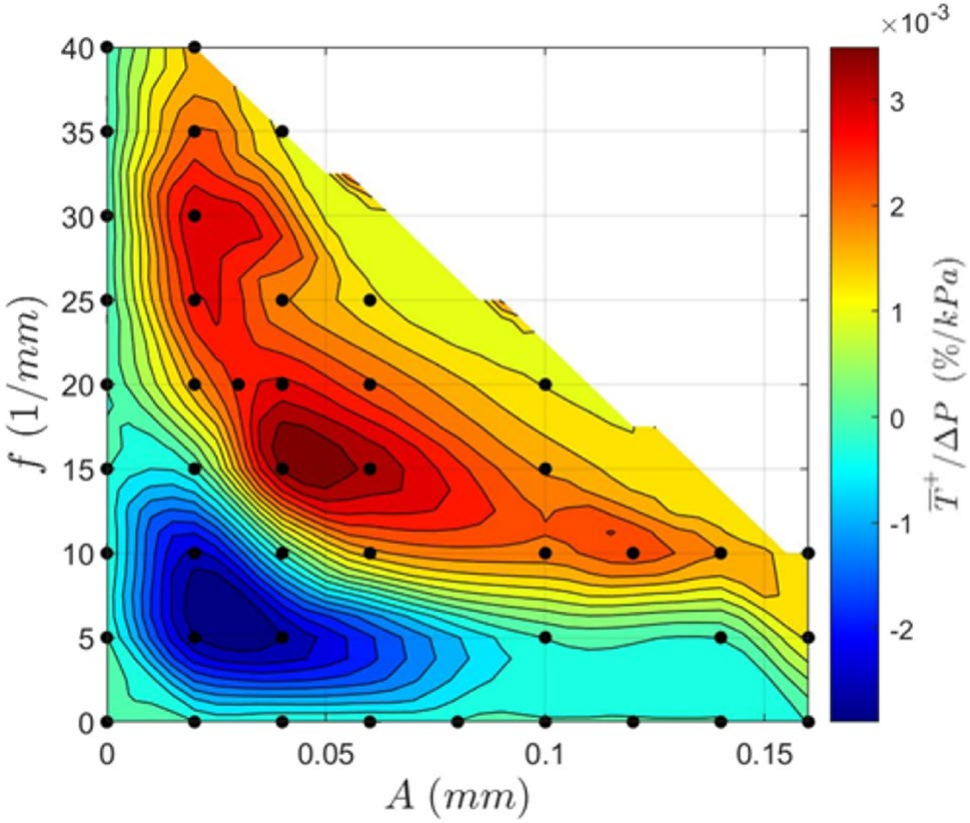
Contour plot showing the average base temperature decrease to pressure drop ratio for a variety of geometries. A high value will exhibit a high-performance efficiency. Re = 580

Another route to find an optimized channel geometry and assess the relationship between the pressure drop and the average base temperature more closely is to consider the ratio of these properties (Fig. 18). Here, a high value for this ratio is desirable since it signifies that the relative cooling gain is greater than the relative pressure drop incurred. It is evident that the highest performance efficiency occurs in the red region at high frequencies and low amplitudes, low frequencies and high amplitudes, or at medium levels of both frequency and amplitude. It is also clear that performance efficiency typically increases with increased amplitude and frequency (at least excluding the blue region of low efficiency) until a maximum is reached, but the efficiency then decreases with increased amplitude and frequency.

**Fig. 19:**
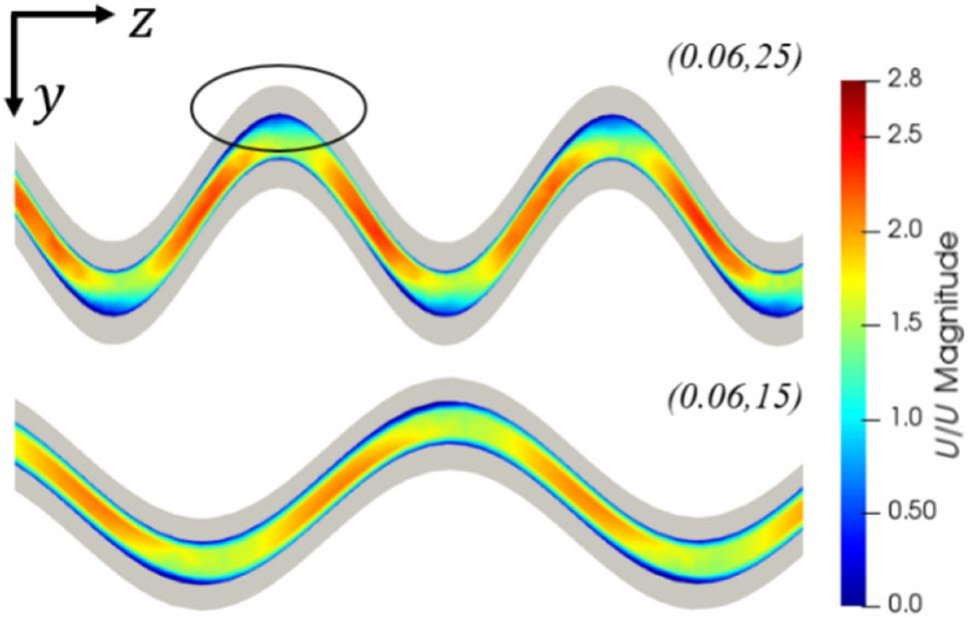
Dimensionless velocity field of geometries (0.06,25) and (0.06,15), showing the onset of flow separation induced by increased spatial frequency. The performance efficiency was reduced due to this separation, despite (0.06,25) exhibiting higher flow velocities. Re = 580

By examining the flow fields for all geometries, it is seen that this reduction in efficiency can be ascribed to flow separation. As the amplitude and frequency increase, the radius of curvature at the inner wall decreases, and at a critical value, the flow begins to separate at the outer wall. The onset of this flow separation is shown in Fig. 19. An example of more extreme flow separation can be seen in Fig. 11. This flow separation contributes significantly to the system losses resulting in a rapid increase in the pressure drop once flow separation has occurred.

Generally, three main factors contribute to the performance and efficiency of the sinusoidal microchannels–convective surface area, flow separation, and the strength of the secondary flow mechanisms.

The convective surface area increases significantly as the amplitude and frequency increase, leading to increased heat transfer. This relates directly to the arc length of the sinusoid, *s*, which can be computed numerically by evaluating the following integral:

10 $$s = \mathop \int \limits_{0}^{L} \sqrt {1 + \left( {Af} \right)^{2} \cos^{2} \left( {fz} \right)} {\text{d}}z$$

where the space coordinate *z* runs down the length of the channel.

Importantly, this is independent of the base area of the channel for these sinusoidal channels. This can be seen by considering the integral describing the base area of the channel, where $\Delta w$ is the channel width:

11 $$\mathop \int \limits_{0}^{L} \left[ {A\sin \left( {fz} \right) + \Delta w} \right] - \left[ {A\sin \left( {fz} \right)} \right] {\text{d}}z = \Delta w L$$

This also shows that the channel number density is independent of amplitude and frequency and thus is purely a function of the channel width and center-to-center channel spacing.

**Fig. 20:**
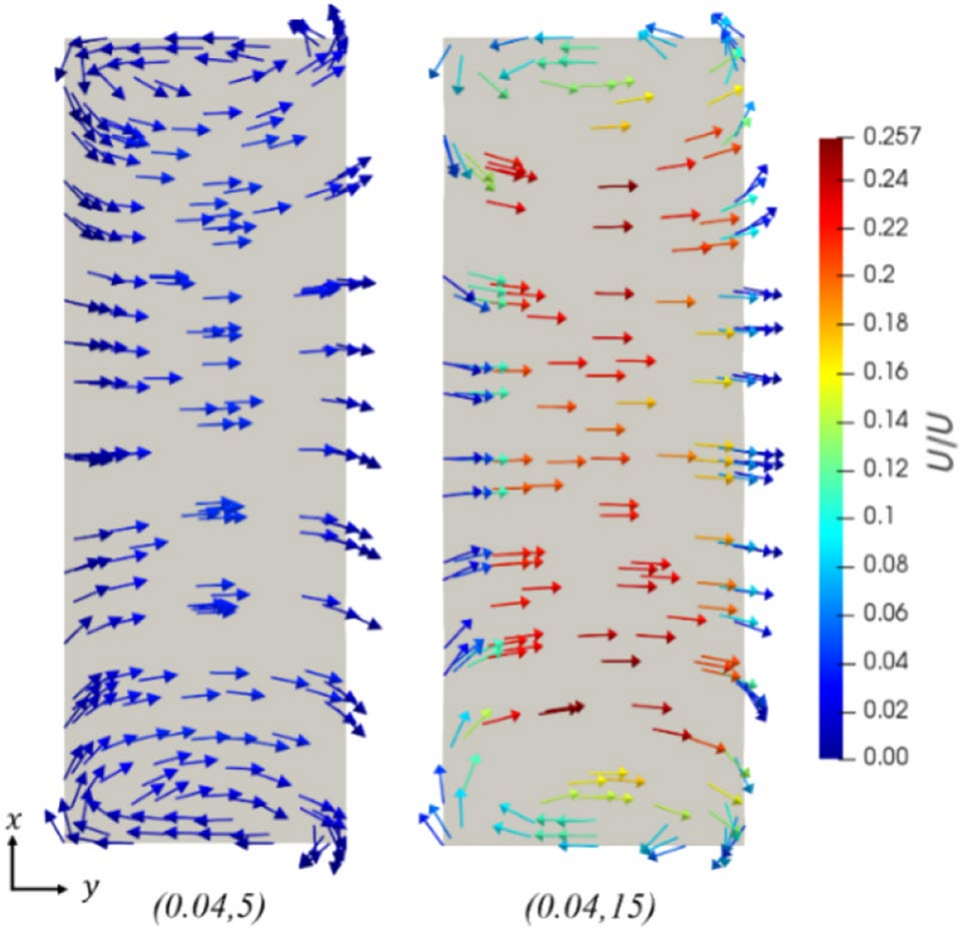
Cross section of the channel, showing the spanwise velocity vector components, colored by their magnitudes, in this case, normalized by the inlet velocity. Dean vortices are observed at the top and bottom of the channel. Re = 580

**Fig. 21:**
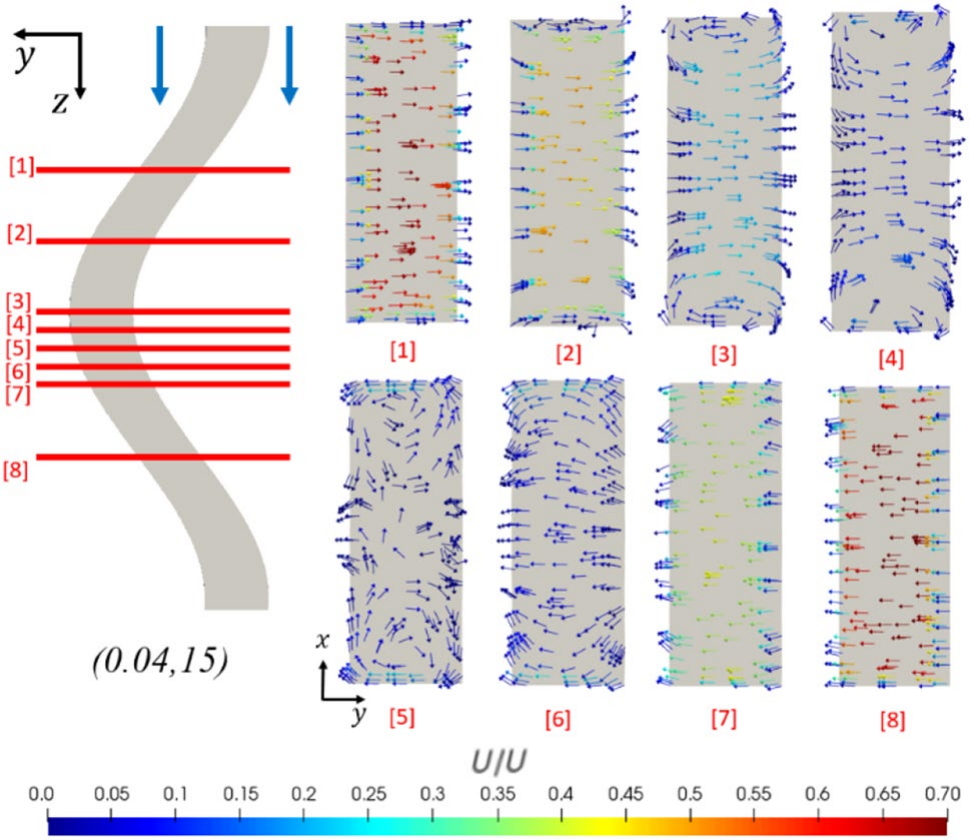
Dimensionless cross-sectional velocity field, with vectors colored by magnitude, shown at successive intervals along the length of the channel. Mixing is seen due to the vortices at the top and bottom of the channel and more aggressively as the flow direction changes in cross section  5. Geometry: (0.04,15). Re = 580

Amplitude and frequency changes also increase the strength of secondary flow mechanisms. In particular, increasing the amplitude and frequency of the sinusoid reduces the radius of curvature at the extrema, giving rise to Dean flow due to the increased centripetal acceleration. Due to these secondary flow mechanisms, this induced rotation within the fluid is seen clearly in the velocity vector fields of Fig. 20 near the top and bottom of the channel. These induced vortices are observed to grow and dissipate as the radius of curvature decreases and increases again, leading to distinct mixing as seen in the cross-sectional velocity vectors in Fig. 21. The strength of these mechanisms is seen to vary significantly with frequency and amplitude, as indicated in Fig. 20, comparing a cross-sectional velocity vector plot for geometries (0.04,5) and (0.04,15). Indeed, an increase in the frequency of the channel by 10 mm^−1^ leads to an increase in the width-wise velocity magnitude by a factor of more than 6, despite the average fluid velocities being the same when streamwise components are additionally considered.

## Conclusions

As the width of straight rectangular microchannels is decreased, large pressure drop penalties are incurred by the system leading to an increased pumping power to achieve the same flow conditions. The pressure drop increases at a rate considerably higher than the temperature-based performance gain, and thus these channels are highly inefficient at widths less than 25 μm at the operating conditions at tested operating conditions. This indicates that decreasing the channel width is often an inefficient design choice, and so other geometric alterations are necessary to address applications with high cooling requirements.

Comparing a selection of microchannel designs that vary geometrically along their length, it was noticed that sinusoidally varying microchannels produced the highest performance among those tested, exhibiting the lowest average base temperatures at the specified operating conditions. This performance can be ascribed to the large convective surface areas, increased thermal energy diffusion rates in regions of flow separation, and increased mixing due to the formation and dissipation of Dean vortices.

Geometric optimization of the sinusoidal channels was undertaken at channel width and height of 35 μm and 100 μm, respectively. Decreases in performance efficiency due to high-pressure drop increase were observed at high channel amplitudes and spatial frequencies where flow separation occurred. Thus, it can be concluded that the highest performance efficiencies are achieved when the channel is at the onset of flow separation and intermediate levels of amplitude and frequency. Based on the developed curves relating the heat flux, pressure drop, and channel geometry, the optimal choice of sinusoidal geometry is calculated at the tested operating conditions for any applied heat flux between 5690 and 6510 kW/m^2^. Unexpectedly, all the optimal geometries in this region had amplitudes approximating 0.04 mm, with increases in frequency being used to increase the heat transfer rate. In a case study of cooling piezoelectric ceramics, the optimized sinusoidal channel reduced the system pressure drop by 79% compared to an equivalent straight channel, when a fixed heat flux requirement was imposed. This was achieved while maintaining a larger minimum feature size, showing the significant benefit of using sinusoidal microchannels and the utility in their optimization.

## List of Symbols

$$A$$: Amplitude, m
$$A_{b}$$: Base area, m^2^
$A_{c}$: Convective surface area, m^2^
$C$: Specific heat capacity, J/kgK
$$D_{H}$$: Hydraulic diameter, m
$$f$$: Spatial frequency, 1/m
$k$: Thermal conductivity, W/mK
$L$: Length of channel, m
$\dot{m}$: Mass flow rate, kg/s
$\mu$: Dynamic viscosity, Pa s
$$P$$: Pressure, Pa
$$\dot{q}$$: Heat flux, W/m^2^
$$Q$$: Heat energy, J
$\dot{Q}$: Rate of heat transfer, W
$$\rho$$: Density, kg/m^3^
$$R$$: Thermal resistance, K/W
$${\text{Re}}$$: Reynolds number
$$t$$: Time, s
$$T$$: Temperature, K
$\overline{T}^{ + }$: Average base temperature decrease, %
$$u$$: Velocity magnitude, m/s
$${\varvec{u}}$$: Velocity vector, m/s
$$U$$: Inlet velocity, m/s
$${\varvec{x}}$$: Position vector, m
